# F58. COMMUNITY-BASED MULTI-SITE RANDOMIZED CONTROLLED TRIAL OF BEHAVIORAL ACTIVATION FOR NEGATIVE SYMPTOMS OF INDIVIDUALS WITH CHRONIC SCHIZOPHRENIA

**DOI:** 10.1093/schbul/sby017.589

**Published:** 2018-04-01

**Authors:** Eunbyeol Lee, Yun-Ji Cha, Ji-Hye Oh, Na-Ri Hwang, Kee-Hong Choi, Ho-Jun Seo

**Affiliations:** 1 Korea University; 2 The Catholic University of Korea

## Abstract

**Background:**

As existing treatments for negative symptoms in schizophrenia have limited empirical support, development of effective treatments for negative symptoms in schizophrenia is in urgent need. Behavioral activation (BA), which is an evidence-based treatment for depression, is a promising candidate treatment for negative symptoms as it proved its feasibility and preliminary efficacy in non-randomized controlled trial for community dwelling individuals with chronic schizophrenia (Choi et al., 2015; Mairs et al., 2011). The primary purpose of the current study was to investigate whether BA would improve negative symptoms as compared with treatment as usual (TAU) for community dwelling individuals with chronic schizophrenia in a multi-site randomized controlled trial. In addition, we explored whether BA would improve other psychiatric symptoms, quality of life and neuro-cognitive functioning.

**Methods:**

For multi-site trials, mental health professionals were trained with BA manual (Choi et al., 2015) and their fidelity was checked by the authors. BA was delivered in a group format once a week for 10 weeks. Participants aged 18 years or older were recruited from community mental health centers and day hospitals in Seoul and Gyeonggi-do area. A total of seventy-two patients with negative symptoms of schizophrenia were randomly assigned into either BA+TAU or TAU. As a primary outcome, negative symptoms were measured using clinical interviews (e.g., BNSS, CAINS, PANSS negative symptoms factor) and self-report questionnaires (i.e., MAP-SR) before and after the 10-week treatments. The secondary outcome measures included other psychiatric symptoms, quality of life, and neuro-cognitive assessment.

**Results:**

BA was well accepted by community dwelling individuals with chronic schizophrenia (drop-out rates of BA+TAU and TAU, 10% and 14%, respectively). Intention-to-treat analyses indicated that compared to TAU condition, BA+TAU group showed greater improvement in negative symptoms, as measured by CAINS, BNSS, and PANSS negative symptom factor (Time*Group interaction effects, F=7.476, p<.01 for CAINS total; F=5.663, p<.05 for BNSS total; F=6.092, p<.05 for PANSS negative symptoms factor). In addition, the results indicated group differences in favor of BA+TAU on the PANSS general psychopathology factor and the Quality of Life, but not neurocognitive functioning (Time*Group interaction effects, F=5.660, p<.05 for PANSS general psychopathology factor; F=7.541, p<.01 for QOL total).

**Discussion:**

The results of the current study demonstrate the feasibility and the efficacy of BA+TAU for negative symptoms of community dwelling individuals with chronic schizophrenia as compared to TAU when delivered by BA trained mental health professionals. Thus, it is speculated that BA is an effective adjunct psychosocial approach to usual comprehensive psychiatric rehabilitation for negative symptoms. Since the current study is ongoing and follow-up data will be available by the time of presentation at SIRS 2018, it will be examined whether benefits of BA would be maintained 3 months after the termination of 10-week BA treatment.

